# Exploring the Prognosis: A Longitudinal Follow-Up Study of Children with Sensory Processing Challenges 8–32 Years Later

**DOI:** 10.3390/children10091474

**Published:** 2023-08-29

**Authors:** Teresa A. May-Benson, Olivia Easterbrooks-Dick, Alison Teasdale

**Affiliations:** 1 TMB Education, Norristown, PA 19401, USA; 2 Spiral Foundation, Newton, MA 02458, USA; oeasterbrooksdick@otathekoomarcenter.com (O.E.-D.); research@thespiralfoundation.org (A.T.); 3 OTA The Koomar Center, Newton, MA 02458, USA

**Keywords:** sensory integration, sensory processing, adults, longitudinal, evidence-based practice, prognosis

## Abstract

Sensory integration and processing challenges have been long recognized in children and, more recently, in adults. To understand the long-term prognosis of these challenges, more research is needed on what children with sensory integration and processing challenges look like as adults. Using the Adult/Adolescent Sensory History, researchers followed up with 102 adults who had known sensory integration and processing challenges as children to examine the following questions: What is the current sensory processing status of adults who received sensory-integration-based occupational therapy services as children? And how has the sensory processing status of adults who received sensory-integration-based services changed since childhood? This study compared performance on sensory processing measures completed as children and as adults for a follow-up group of adults. The results revealed that the severity of sensory integration and processing challenges experienced by the follow-up group decreased from childhood, with 51% of the follow-up group now scoring in the “typical” range of sensory processing. Our findings suggest that those children with sensory integration and processing challenges who are recognized and seek occupational therapy services using an ASI approach are likely to have a good long-term prognosis regarding the severity of their sensory processing functioning.

## 1. Introduction

Sensory integration intervention was developed by occupational therapist A. Jean Ayres over 50 years ago to address sensory–motor challenges in children with learning disorders [1]. Patterns of sensory integration and processing challenges have been identified in children [2,3], along with the development of valid and reliable assessments to identify these patterns [2,4,5,6]. Recent research indicates that sensory integration and processing challenges in children impact occupational performance [7,8], academic achievement and learning [1,9], behavior [10,11,12], and mental health [13]. Since these patterns and their impact on performance have been recognized, the Ayres Sensory Integration™ (ASI) intervention has become one of the most recognized and utilized interventions for children with challenges processing and integrating sensations [14,15].

Occupational therapists using ASI are concerned with providing evidence-based interventions in their practice [16,17]. Evidence-based practice (EBP) consists of combining the best research evidence available with a clinician’s experience and the client’s desires and experiences to inform and guide clinical decision making [16] about the provision of therapeutic services [18]. To engage in EBP, research evidence needs to describe the clinical population; support the use of valid and reliable assessments; examine the prevalence, etiology, and diagnosis of the condition; and determine the effectiveness and cost–benefits of available interventions for the condition [19]. Lastly, research evidence is needed to examine the prognosis associated with the condition, including the long-term health risks/benefits, the outcomes of the condition, and the interventions used to treat it. Lamb and Metzler (2014) [17] further stated that evidence-based practice should reflect policy implications. They emphasized the importance of linking the value of occupational therapy to the priorities of the healthcare system. For practitioners working with children with sensory integration and processing challenges, it is important to discover how this presentation manifests over the lifespan, identify lifelong sensory and occupational profiles, and assess long-term health outcomes. This information has far-reaching implications for policy decisions, including early intervention access and insurance reimbursement.

Currently, most of the research on sensory integration and processing is focused on a single time point in childhood or short periods of follow-up time. Limited research exists that investigates the long-term experiences of individuals with sensory integration and processing difficulties from childhood into adulthood to inform policy decisions and occupational therapy practitioners’ clinical practice. Research examining sensory integration and processing in adults with no additional diagnoses is even more limited. To develop an understanding of the progression of sensory integration and processing challenges, stakeholders must be aware of this presentation in childhood, adulthood, and over time.

Several studies have examined the relationship of children’s sensory integration and processing patterns with behavior, occupational performance, and academics during childhood. DeGangi, Porges, Sickle, and Greenspan (1993) [20] found that untreated infants with moderate-to-severe sensory regulatory challenges at 8–11 months had developmental, sensory–motor, and/or emotional and behavioral deficits at 4 years of age. Ben-Sasson, Carter, and Briggs-Gowen (2009) [21] found that early sensory over-responsivity in 1–3-year-olds predicted later sensory over-responsivity in elementary school at 7–10 years of age. They suggested there was a strong relationship between sensory processing challenges and other comorbid mental health diagnoses in children [21]. Similarly, these researchers found that parents of school-aged children with sensory over-responsivity reported that their children had more frequent internalizing, externalizing, and dysregulation behaviors, as well as lower levels of adaptive social behaviors than their non-over-responsive peers [21]. Lastly, Parham (1998) [22] examined the relationship between sensory integration and processing functioning and the academic skills of arithmetic and reading in children 6–8 years of age. She found that sensory integration and processing functioning were strongly related to arithmetic at 6–8 years of age, but the strength of the relationship decreased with age over the four years of follow-up. However, reading was not related to early sensory integration and processing function but was related to it four years later. Praxis skills were found to be specifically related to arithmetic at follow-up [22].

The emphasis in the literature on sensory integration and processing has been on children. However, in recent years, sensory integration and processing challenges have been identified and examined in typically functioning adults and adults with a variety of comorbid diagnoses, including attention-deficit/hyperactivity disorder (ADHD) [23,24], anxiety [25,26], autism [23], depression [27], and other mental health conditions [28,29]. As with children, the emphasis of most studies on adults with sensory integration and processing challenges has focused on sensory defensiveness/over-responsivity or sensory modulation challenges. Research revealed that these sensory challenges in adults are related to numerous functional life, health, and quality of life issues [30,31,32]. A study of college students with sensory integration, processing difficulties, and ADHD reported that sensory issues negatively impacted their choice of study and exam environments, leisure skills, and social engagement [23]. Similarly, another study concluded that university students’ sensory integration and processing challenges were related to interpersonal problems [33]. In addition, in a recent study by McMahon, Anand, Morris-Jones, and Rosenthal (2020) [34], which asked adults about their current and childhood sensory experiences, it was found that high symptoms of sensory integration and processing challenges in childhood may lead to high symptoms in adulthood. Finally, adults who had identified sensory integration and processing challenges as children reported having decreased quality of life if their sensory–motor challenges were still prevalent in adulthood in comparison to adults who had sensory integration and processing challenges in childhood and reported current functional sensory–motor profiles [35].

The existing literature supports the presence of challenges in processing and integrating sensation in adults and the relation of these sensory patterns with functional life performance. Research on both children and adults supports a relationship between sensory over-responsivity or sensory modulation challenges and anxiety. However, except for McMahon et al. (2020) [34], only current adult sensory processing status has been studied [36]. How sensory characteristics change from childhood to adulthood is unknown. To date, studies of sensory integration and processing in adults have not specified if those adults had these sensory patterns as children or whether they received occupational therapy intervention. Thus, there are no known studies on the long-term presentation of sensory integration and processing challenges in children who are now adults. There is also no information on the long-term prognosis of sensory integration and processing challenges in children who seek ASI intervention. Long-term health outcomes of childhood challenges in processing and integrating sensation and the influence of ASI intervention on those outcomes are needed to fully engage in evidence-based practice and support practitioner clinical reasoning and policy development. In addition, as noted, studies have focused primarily on children with specific diagnoses such as ADHD, autism, and DCD; however, the long-term outcomes of children with no diagnoses other than sensory integration and processing challenges have not been examined.

The purpose of this study was to gather preliminary information on long-term presentation and change in sensory integration and processing, as well as motor functioning, in an adult population with identified sensory integration and processing difficulties and no other diagnoses at intake as children. This study was a descriptive longitudinal follow-up study that consisted of identifying individuals who sought sensory-integration-based occupational therapy services as children and who participated in an online survey of their current sensory processing skills as adults 5–32 years later. This study is not intended to be an intervention efficacy study but an examination of the current adult status of children with sensory integration and processing challenges, which may inform the prognosis of these children.

This study examined the following research questions:

What is the current sensory processing status of adults who received sensory-integration-based occupational therapy services as children 5–32 years later?
How has the sensory processing status of adults who received sensory-integration-based occupational therapy services as children changed since childhood?

## 2. Method

The SPIRAL Foundation Institutional Review Board approved this study on 8 January 2019 for protocol #1039. All adult participants provided informed consent as well as permission to access historical client records following the Declaration of Helsinki. Deidentified data from non-responders were collected according to recommendations of the Office of Human Rights Protection. Privacy protection for personal health information was implemented according to HIPAA.

### 2.1. Participants

Potential participants were children who sought sensory-integration-based occupational therapy services at a private occupational therapy practice based in the northeast United States between 1983 and 2013. The inclusion criteria were as follows: At the time of initial service, the child was <18 years of age; had no reported diagnoses except sensory processing challenges at intake; the practice’s sensory history was completed by the parent and/or adolescent; was aged 18–50 years at the time of adult survey completion; and had a service discharge date prior to 2014. The private practice divided archived clinical records into five groups based on five-year discharge periods. A stratified blocked random sampling method was used to select potential individuals from each discharge period to participate in the follow-up survey. A total of 1272 individuals received invitations to participate in the study.

#### 2.1.1. Longitudinal Follow-Up Group

In total, *N* = 102 participants completed all or part of a multi-part online survey. At the time of initial services, this group had *M* = 6.9 years and *SD* = 3.0 years of age. At follow-up, this group had *M* = 27.2 years and *SD* = 5.8 years of age. See Table 1 for the demographics of participants at the time of initial services.

#### 2.1.2. Longitudinal Non-Responder Group

Deidentified data from the clinical records of 50 individuals who did not respond to the invitation to participate in this study were included to examine potential differences or biases between responders and non-responders. The private practice randomly selected 10 participants from the clinical records of each of the five discharge year blocks for inclusion. The adult age of non-responders was calculated by determining age on a set date identified within the study data collection period, which resulted in a group mean adult age of 27 years, (*SD* = 6.4 years). At the time of initial services, this group had a childhood age of *M* = 7.0 years and *SD* = 2.6 years. See Table 1 for demographics.

### 2.2. Measures

Two self- or parent-reported measures of sensory processing were utilized. The child measure was the OTA the Koomar Center Sensory History (SXHX) [37], which was obtained from the participants’ clinical records. The adult measure was the Adult/Adolescent Sensory History [38], which was collected as part of a multi-part online survey, which also included an informed consent document and demographics (e.g., age, education, employment, current diagnoses, etc.).

#### 2.2.1. Adult/Adolescent Sensory History (ASH)

The ASH utilizes the Ayres Sensory Integration^®^ model of sensory processing and is a standardized self-report questionnaire consisting of 163 items. This measure assesses behaviors believed to represent sensory modulation, sensory discrimination, praxis–posture, and social–emotional functioning. It was developed from a clinical adult version of the OTA the Koomar Center Sensory History (SXHX). The ASH was formatted as part of an online survey for this study within Qualtrics (Qualtrics, Provo, UT, USA, https://www.qualtrics.com), a secure and HIPAA-compliant survey platform. Items are rated on a 5-point Likert scale (1 = Never, 2 = Rarely, 3 = Sometimes, 4 = Often, and 5 = Always). The results are presented in standard *z*-scores, with high scores indicating more dysfunction. The ASH assesses functioning in the following areas: visual–spatial, auditory–language, movement, taste–smell, touch, proprioception processing, postural control, praxis skills, and social–emotional functioning. The test results in a total score, with individual subscores for each sensory processing/motor/social area, sensory modulation, and sensory discrimination functions. Scores are reported based on typical (scores up to +1.0 SD), mild (scores of +1.0 to +2.0 SD), and definite (scores greater than +2.0 SD) difficulty categorization. The validation of scores was carried out through factor and Rasch analysis of items (May-Benson, 2015) [38]. The total score and sensory sections had a good internal consistency of 0.80–0.97 using Cronbach’s coefficient alpha. The inter-rater (*r* = 0.68 for the total score and 0.39–0.77 for subscores) and test–retest reliability (*r* = 0.85 for the total score and 0.74–0.87 for subscores) were good (May-Benson, 2015) [38].

#### 2.2.2. OTA the Koomar Center Sensory History (SXHX)

The OTA the Koomar Center Sensory History (SXHX) was developed using the same ASI framework at the same private practice as the original clinical version of the ASH. The SXHX consists of several versions of a parent- or self-reported clinical sensory–motor history measure used in a sensory-integration-based occupational therapy private practice to provide detailed information on sensory processing, motor skills, and social–emotional functioning of children and adolescents. Different versions of the measure are utilized for individuals from infancy to 18 years of age. Each version of the SXHX includes sensory–motor questions, comparable to the ASH, in the areas of visual–spatial, auditory–language, movement, taste and smell processing, tactile processing, motor control, and social–emotional functioning. A 3-point Likert scale (1 = Never/Rarely, 2 = Sometimes, and 3 = Often/Always) was used for all the ratings on this measure. This clinical measure is not standardized. Because childhood clinical data included varying SXHX versions with different numbers of items in various sections and missing data, the means of item raw scores were calculated for the total score and all section scores on the 1–3 scale of the SXHX. The items used from the SXHX for this sample were in the range of *N* = 26–149 (*M* = 117, *SD* = 14.4). This measure was used as it was the only assessment tool that was consistently collected from all clients at intake at this clinical facility. There were no other consistent measures of sensory processing available.

### 2.3. Procedures

This follow-up study proceeded in several steps.

#### 2.3.1. Step 1: Recruitment

Staff from the clinical private practice completed Step 1 according to HIPAA guidelines and regulations. The archived clinical records of the private practice (organized by discharge year) were divided into five blocks of participants, each consisting of a 5-year time frame. The first block included clients with discharge dates 5–10 years before the start of the study and proceeded to the last block, which included individuals who were discharged from services >25 years prior to the study. Within each block, the list of discharged clients was randomized to determine the order in which individuals would be invited to participate in the study. Over 10 months, records were examined for adherence to eligibility criteria (e.g., the presence of the OTA Sensory History, the absence of diagnoses, etc.). Records were removed if they did not meet inclusion criteria. For records that met inclusion criteria, the names, addresses, and phone numbers of the clients and their parents were recorded. Publicly available internet information (e.g., white pages) was used to update the parent and child’s contact information. Invitation letters from the private practice were sent to the now-adult child. This letter invited the previous clients to participate in a research study conducted by the SPIRAL Foundation. Where possible, the same number of individuals from each discharge block were contacted; however, this was not always possible as some blocks had a relatively low number of individuals meeting the study inclusion criteria. Recruitment ultimately resulted in 1272 individuals being invited to participate in the research. A total of 117 invitations were returned by the post office as undeliverable.

#### 2.3.2. Step 2: Data Collection

The invitation letter, sent to all potential participants, contained a link to the multi-part online survey, which was accessed by 113 individuals (9.8% response rate of non-returned invitations). Contact information for both the private practice and the research facility was provided in the event individuals desired to be removed from the study contact list or had questions about the study. Eleven individuals (1.0%) contacted the private practice or study personnel to request removal from the mailing list. Up to two additional reminder letters were mailed to the potential participants. Active recruitment continued over ten months; however, individuals continued to respond for a further eight months. The study had a final response rate of 8.8% (*N* = 102) of eligible individuals who completed at least one section of the ASH. An informed consent document, including permission to access childhood clinical records for analysis, was completed by all participants along with the demographics form and the ASH.

#### 2.3.3. Step 3: Clinical Data

The private practice provided researchers with the deidentified clinical data of the 50 randomly selected non-responders (*n* = 10 per discharge year group) and the identified clinical data of all participants who completed the informed consent. The participants’ clinical and survey data were matched using both name and birth date.

#### 2.3.4. Step 4: Data Transformation

The SXHX and the ASH total and subsection scores measure the same underlying concepts of Ayres Sensory Integration; however, item scales are 1–3 for the SXHX and 1–5 for the ASH. In addition, not all items are identical, and the number of items for the total and section scores differs. To compare the child and adult scores, raw scores on the ASH were transformed to a 3-point scale consistent with the SXHX. First, individual item scores on the ASH were recoded from the 5-point scale to the 3-point scale of the SXHX. The labeling of the score benchmarks allowed this transformation to be accurately completed. For the ASH, the motor control section was defined as including all items from the proprioception, postural control, and praxis sections consistent with the SXHX measures. The mean item scores were then calculated on the transformed responses for comparison with childhood data for the total score and the following sections: visual–spatial, auditory–language, movement, taste–smell processing, tactile processing, motor control, and social–emotional functioning.

In the analyses that included only adult data, raw scores or standardized category classifications were used based on the 5-point item responses. In the analyses that included childhood data or compared child and adult data, the mean total and section raw scores were used based on the 3-point item responses.

### 2.4. Data Analysis

Demographic data were completed using means, frequency counts, standard deviations, and percentages. The approximate duration of occupational therapy services at the private practice was calculated by subtracting the year of intake from the year of discharge. The ASH standardized score category classifications of typical, mild, and definite difficulties described the participants’ current functioning level.

Chi-square and independent-sample *t*-tests were used to examine the differences between the childhood data of the follow-up group and the non-responder group. Uni- and multivariate analyses of covariance were used to examine the differences in current sensory processing by various demographic variables.

Correlational analyses were used to examine the relationships between the child and adult total and sectional mean raw scores, and paired *t*-tests were used to examine the differences between the reported severity of sensory processing challenges from childhood to adulthood. All analyses were conducted using SPSS v. 22 (IBM Corp. Released 2013. IBM SPSS Statistics for Windows, Version 22.0. Armonk, NY, USA: IBM Corp.).

## 3. Results

### 3.1. Demographics of Follow-Up Group

The 102 respondents (males, *n* = 65, females *n* = 37) who completed the follow-up survey had a mean age of *M* = 27 years, with *SD* = 5.8 years (range = 18–40 years). The follow-up period was *M* = 21 years, with *SD* = 5.6 years (range = 8–33 years).

#### 3.1.1. Current Diagnoses

At intake as children, none of the participants had any diagnoses other than suspected sensory integration challenges requiring referral for an occupational therapy evaluation or services. At follow-up, 86 respondents shared diagnostic information. Of those, 88% reported one or more current diagnoses other than sensory integration and processing challenges (*m* = 2.9 diagnoses), e.g., ADHD, anxiety, ASD, depression, learning disabilities (LD), or others. Notably, 51% of those reporting diagnostic information *(n* = 44) had self-reported anxiety, with a total of *m* = 4.4 comorbid diagnoses reported in those with anxiety. Those with self-reported anxiety accounted for *n* = 19 of the 25 (76%) participants with reported LD and *n* = 28 of the 36 (78%) participants with reported depression.

#### 3.1.2. Education and Employment

Most participants reported that they were either employed full-time (51%, *n* = 52), were full-time students (31%, *n* = 32), or were both (2%, *n* = 2). Others reported they were either part-time employed, were part-time students, worked at home, were seeking employment, were disabled, or were a combination of these descriptions. Of the 63 participants who were not students, 79% (*n* = 49) reported having bachelor’s degrees or higher, with 27% (*n* = 17) reporting post-graduate degrees and 8% (*n* = 5) having doctoral degrees. Of the remaining 38 participants who were students, 37% (*n* = 14) had already completed at least a bachelor’s degree.

#### 3.1.3. Current Services

Five (5%) individuals reported that they were currently receiving sensory-integration-based occupational therapy intervention services as adults. Notably, 44 (43%) individuals reported currently receiving psychological services, and an additional 18 (18%) respondents reported having received psychological services in the past. Of these 62 participants who reported currently or previously receiving psychological services, 40 (65%) had self-reported anxiety.

### 3.2. Comparison between the Follow-Up Group and Non-Responders

The follow-up group was similar to the non-responder sample in age at intake; however, chi-square (χ^2^ (1, *N* = 152) = 5.3, *p* = 0.021) results revealed that there were significantly fewer females in the non-responder group (18%) than in the follow-up group (36%). Responses were received from potential participants in each of the discharge periods; however, clients discharged 15–19 years ago were more likely to respond, whereas those discharged 25–30 years ago were less likely to respond. A two-sample independent *t*-test was non-significant for the differences in the length of intervention between groups (responders *M* = 1.5 years, with *SD* = 1.85 years, and non-responder *M* = 1.5 years, with *SD* = 2.13 years). In the independent two-sample *t*-tests, no significant differences were observed in the average raw item scores for the SXHX as children between responders and non-responders in terms of the total score and all section scores, except for the Motor section, where statistically significant differences were found (*t* (150) = 2.1, *p* = 0.039). Responders (*M* = 1.8, *SD* = 0.39) had more difficulties than non-responders (*M* = 1.6, *SD* = 0.32). The effect size between groups for the motor section was *d* = 0.57. See Table 1 for specific details. Based on these data, the follow-up group appeared to be a representative sample of previous clients.

### 3.3. Current Sensory Processing

The current sensory processing of the follow-up group as adults was determined based on their responses to the ASH. The group means for the ASH total score and all major subscores, except social–emotional, were in the typical range as defined by the standard scores on the ASH. Overall, 50% of the follow-up group had typical total ASH scores, 30% had mild difficulties, and 20% had definite difficulties as adults. In contrast, of those who had self-reported anxiety, 27% scored in the typical range, 43% fell under the mild difficulty category, and 30% were in the definite difficulty category. This is compared to the 64% who scored in the typical category, 24% in the mild difficulty category, and 12% in the definite difficulty category among those not reporting anxiety and the 79% who scored in the typical range, 7% in the mild difficulty range, and 14% in the definite difficulty range among those who did not report diagnostic information. Thus, self-reported anxiety was associated with more severe sensory processing challenges (*t* (84) = −3.6, *p* = 0.001). ASH scores were in the typical range for 52–72% of the participants depending on section subscores. See Table 2 for specific subscores.

ANCOVA (*F*(5, 80) = 6.7, *p* < 0.001) revealed no significant differences in terms of gender, age, anxiety, or currently receiving psychological services in the ASH total score; however, the number of reported diagnoses was highly significant (*F*(1, 80) = 9.6, *p* = 0.003), with those having more diagnoses reporting more difficulties with sensory processing. MANCOVA results for five sensory, three motor, and one social–emotional sections, with the same independent variables, were statistically significant only for gender (*F*(9, 72) = 2.3, *p* = 0.027, Wilks’Λ = 0.779, *η*^2^ = 0.221) (Table 3). Follow-up ANCOVAs revealed that there were statistically significant differences between genders (*F*(1, 80) = 4.8, *p* = 0.032, *d* = 0.72) only in the movement section, with females reporting more problems in this area (*M* = 61.2, *SD* = 13.7) than males (*M* = 50.8, *SD* = 15.3). For the modulation and discrimination sections, the number of reported diagnoses was statistically significant (*F*(2, 78) = 4.7, *p* = 0.009, Wilks’Λ = 0.887, *η*^2^ = 0.104). Follow-up analyses revealed that those reporting more diagnoses also had statistically more difficulties in discrimination (*F*(1, 79) = 10.1, *p* = 0.002) and modulation (*F*(1, 79) = 4.8, *p* = 0.032).

### 3.4. Change in Sensory Processing from Childhood to Adulthood

The paired *t*-tests on the mean total raw score and all section scores (using the three-point scale) revealed a statistically significant decrease in reported sensory processing difficulties from childhood to adult follow-up in all areas except auditory processing, which approached significance (Table 4). In a *t*-test, the childhood total score was *m* = 1.7, with *sd* = 0.23, and the adult score was *m* = 1.5, with *sd* = 0.31 (*t* (96) = 5.9, *p* < 0.001 (*d* = 0.74)), thus reflecting a large significant difference in the overall decrease in the severity of the reported sensory symptoms from childhood to adulthood.

Pearson correlations were computed to further examine the relationship between the total, sensory, motor, and social–emotional section scores in childhood and adulthood. Auditory processing scores in childhood had small statistically significant relationships to auditory processing (*r*^2^ = 0.29, *p* = 0.003), visual–spatial processing (*r*^2^ = 0.28, *p* = 0.005), tactile (*r*^2^ = 0.21, *p* = 0.036), social–emotional (*r*^2^ = 0.22, *p* = 0.034), and total scores (*r*^2^ = 0.25, *p* = 0.014) in adulthood. In addition, auditory processing in adulthood had statistically significant relationships to tactile processing (*r*^2^ = 0.22, *p* = 0.026), social–emotional (*r*^2^ = 0.27, *p* = 0.005), and total scores (*r*^2^ = 0.24, *p* = 0.018) in childhood. A few additional very small (*r* < 0.20) significant relationships were also sporadically found, which did not appear to be clinically meaningful.

## 4. Discussion

To the authors’ knowledge, this study is the first to follow up and examine current sensory processing in adults with known challenges in processing and integrating sensation as children. The results of this study provide information on the long-term prognosis and adult functioning of children with sensory processing concerns who received ASI services. This study does not specifically examine intervention efficacy but investigates a specific adult population with a history of sensory challenges who received services at a single occupational therapy practice, which may inform clinicians and policymakers about the possible long-term presentation of sensory processing characteristics in children with sensory processing and integration challenges.

Our results reveal that, overall, children with known challenges processing and integrating sensation who receive ASI services are likely to perform in the typical range of sensory processing functioning as adults. In addition, the severity of sensory processing challenges significantly decreased over time in this study population, with 50% of participants performing in the typical range as adults. Only 27% of the adult participants reported definite difficulties in overall sensory processing. Interestingly, only auditory processing persisted as a notable problem area into adulthood, demonstrating no significant change from childhood to adulthood. In addition, auditory processing in both childhood and adulthood was significantly related to tactile processing, social–emotional processing, and total scores. It is possible there was little change in this area as it is a sensory system that is not routinely treated by sensory-integration-trained occupational therapists even though auditory defensiveness is routinely observed clinically with tactile defensiveness. It is hypothesized that challenges in auditory and tactile processing may contribute to social–emotional challenges later in life, which may result in the increased anxiety found in these adults. Regardless, these findings suggest that auditory processing is important for occupational therapists to address, not only for sensory reasons but also to potentially promote social–emotional well-being.

Previous research revealed that children with challenges processing and integrating sensations were likely to have multiple comorbid diagnoses [21,39]. Particularly common among these diagnoses were learning challenges, anxiety, and depression. This suggests that children with sensory processing issues may be particularly vulnerable to learning and mental health challenges. Tzang et al. (2019) [39] found that Taiwanese children with ADHD who received sensory integration intervention were likely to have 3.5 times as many diagnoses as children with ADHD who did not receive sensory integration intervention. A more detailed examination of the possible implications of these comorbid diagnoses and the potential role of interventions for these other diagnoses on changes in sensory processing should be examined in future studies.

McMahon et al. (2020) [34] found that children and adults with challenges processing and integrating sensations were likely to have similar difficulties in sensory–motor functioning as well as problems with self-regulation and anxiety. The results of this current study concurred with McMahon et al. (2020) in that 88% of the respondents who reported diagnostic information had an average of 2.9 comorbid diagnoses. However, the participants in our follow-up group had fewer comorbid diagnoses than the study by Tzang et al. (2019) [39], which focused on children with ADHD. Over 51% of participants in our follow-up group with diagnoses reported having anxiety, making anxiety the most frequently reported diagnosis in this adult population. This result is consistent with the findings of McMahon et al. (2020) [34] regarding anxiety in adults with childhood sensory processing challenges. Furthermore, McMahon et al. (2020) [34] suggested that early challenges in processing and integrating sensations may contribute to later anxiety, which is consistent with the findings of this study. Carpenter et al. (2019) [40] found that preschool childhood sensory hyper-responsivity unilaterally predicted school-age anxiety, thus establishing a clear relationship between sensory processing challenges and anxiety. Panchyshyn et al. (2023) [41] suggested that intolerance to the uncertainty caused by sensory defensiveness could play a vital role in causing increased anxiety, especially in women. Therefore, the mechanism of the relationship between sensory processing and anxiety is unknown, but some clinicians hypothesize that sensory processing challenges result in trauma, which is perhaps reflected in the uncertainty suggested by Panchyshyn et al. [41], to the developing nervous system, which can then result in anxiety. This causal relationship should be examined in future studies.

In this follow-up study, individuals with anxiety were the most likely to seek psychological services. It is unknown if individuals with anxiety and sensory processing and integration challenges are more likely to complete a survey such as this to gain insight into their functioning, resulting in this high group number. In this study, while there was no difference in sensory processing of children among those who did and did not have later anxiety, at follow-up, adults with anxiety had more severe sensory processing challenges than those without anxiety and were more likely to perform in the mild or definite difficulty range of sensory processing. Consistent with the findings of McMahon et al. (2020) [34], this study further suggests that children who receive early occupational therapy services for challenges in processing and integrating sensations demonstrate a decreased severity of sensory processing challenges in adulthood.

Furthermore, despite the potential later mental health and learning diagnoses, children with challenges processing and integrating sensations who received occupational therapy services reported functional occupational performance through full-time employment and participation in full-time educational activities at follow-up. The participants in this study were highly educated with the majority having at least a bachelor’s degree and over 25% having master’s or doctoral degrees, and many of the remaining individuals were pursuing post-graduate education. The parents at the private practice where this study was conducted were also generally highly educated, and it is possible that those who are more highly educated are more likely to respond to this survey and agree to participate in research activities. Furthermore, children with other diverse backgrounds and who receive services at other facilities may have different results. Thus, our findings may be limited to this study population.

The clinical sample of children with sensory processing and integration challenges at this study facility typically consisted of approximately 73% percent males and 27% percent females (May-Benson et al., 2009) [42]. The rate of responders in this study was similar, at 68% male and 36% female, thus reflecting the general clinical population. However, in this study, the randomly selected non-responder sample found that women constituted a much lower percentage of the non-responder child population than expected. Therefore, although our clinical facility typically had more males, and our non-respondents had slightly fewer females than expected, this difference had no significant impact on our findings other than on movement problems. Women responders demonstrated more significant movement challenges than the male responder group. This is possibly due to women clinically presenting with more gravitational insecurity than men (May-Benson et al., 2016) [43]. This is also consistent with the increased prevalence of movement and balance problems in autistic females over males reported by Osorio et al. (2021) [44]. Other than the movement scores, there were no differences between those participants who responded and those who did not in terms of childhood demographics or their sensory processing difficulties. Furthermore, another study investigating this same study group found that the follow-up group who reported typical sensory–motor processing as adults demonstrated a high level of quality of life, although those who reported difficulties in sensory–motor functioning had challenges in the physical health aspect of quality of life [35].

### Study Limitations

This study presents exciting and novel insight into the long-term prognosis of children with challenges processing and integrating sensation who receive early sensory-integration-based occupational therapy services. This study involves the longest follow-up period yet identified in the literature. It is recognized, however, that this study has several limitations. First, this study involved a retrospective review of childhood clinical records, which resulted in the use of several variations of the sensory history assessment. This required some data conversion to allow for comparison across measures. In addition, the childhood versions of the sensory history assessment were not standardized, while the adult version was. Although both versions were developed by the same individuals and used in the same facility, this difference should be considered in interpreting the results. Secondly, childhood sensory histories were largely completed by parents, while adult sensory histories were self-reported. Previous studies on ASH suggest that parent reports underestimate the severity of sensory processing in children [38]; therefore, changes from childhood to adulthood may have been even greater. Lastly, this follow-up study may involve a self-selection bias in which specific individuals are likely to respond to a study like this (e.g., more educated individuals). Data on non-responders do not suggest significant differences in childhood, but it is possible that those with more or less severe issues may have chosen not to respond as adults. Furthermore, this study sample consisted of a very homogenous population that was largely white and in the middle to upper-middle socio-economic status at a specific occupational therapy practice.

Future studies examining sensory processing challenges in adults should document if participants were identified as children with challenges processing and integrating sensation and if early sensory-integration-based occupational therapy services were provided. Long-term prospective follow-up studies are needed to confirm the findings of this study. In addition, the functioning of this population in daily life in terms of occupational performance should be examined.

## 5. Conclusions

### Clinical Implications

This study provides preliminary information on the long-term prognosis of children with challenges processing and integrating sensations who receive ASI services. Our findings suggest that, as adults, these children are likely to have average sensory–motor performance, engage in successful educational activities, and be gainfully and successfully employed. Our findings provide preliminary support for childhood sensory-integration-based services to address challenges with processing and integrating sensation.

## Figures and Tables

**Table 1:** Comparison of demographic and sensory history information for the follow-up group and non-responders.

Demographic	Follow-Up Group	Non-Responder Group
Gender Male Female	*n* = 65 (64%) *n* = 37 (36%)	*n* = 41 (82%) *n* = 9 (18%)
Intake Age	*M* = 6.9 years *SD* = 3.0 years Range 1.4–16.0 years	*M* = 7.0 years *SD* = 2.6 years Range 1.9–12.10 years
Length of Services Up to 1 year 1–2 years 3+ years	*n* = 40 (39%) *n* = 38 (37%) *n* = 24 (24%)	*n* = 21 (42%) *n* = 16 (32%) *n* = 13 (26%)
Discharge Year 2009–2013 2004–2008 1999–2003 1994–1998 1989–1993	Responders *n* = 6 (7%) *n* = 24 (8%) *n* = 34 (12%) *n* = 24 (8%) *n* = 15 (5%)	Non-responders *n* = 78 (93%) *n* = 296 (92%) *n* = 261 (88%) *n* = 269 (92%) *n* = 266 (95%)
Child Mean Score on SXHX Total Visual–Spatial Auditory Movement Taste and Smell Tactile Motor Social–Emotional	*M* = 1.7 *SD* = 0.23 *M* = 1.6 SD = 0.36 *M* = 1.9 SD = 0.50 *M* = 1.7 SD = 0.25 *M* = 1.6 SD = 0.44 *M* = 1.7 SD = 0.35 *M* = 1.8 SD = 0.39 *M* = 1.9 SD = 0.31	*M* = 1.7 SD = 0.24 *M* = 1.6 SD = 0.75 *M* = 1.7 SD = 0.39 *M* = 1.7 SD = 0.24 *M* = 1.5 SD = 0.44 *M* = 1.6 SD = 0.39 *M* = 1.6 SD = 0.32 * M = 1.9 SD = 0.38
Current Age	*M* = 28 years *SD* = 5.8 years Range = 18–40 years	*M* = 27 years *SD* = 6.4 years Range = 18–41 years
Follow-Up Period	*M* = 20 years *SD* = 5.6 years Range = 9–33 years	*M* = 18 years *SD* = 7.3 years Range = 6–32 years

* Independent samples’ *t*-test was statistically significant *t* (150) = 2.09, *p* = 0.039; all other comparisons were statistically non-significant.

**Table 2:** ASH mean raw and *z*-scores and percentages of adult participants’ scores within each test category.

ASH Scores	*N*	Mean Raw Score (*SD*)	Mean *z*-Score	Typical % (*n*)	Mild % (*n*)	Definite % (*n*)
Total	99	349 (88.3)	−0.99	50 (50)	30 (30)	20 (19)
Visual	101	55 (16.2)	−0.73	57 (57)	32 (32)	13 (12)
Auditory	101	31 (11.0)	−0.64	64 (64)	17 (17)	21 (20)
Movement	100	55 (15.5)	−0.47	72 (72)	22 (22)	7 (7)
Taste and Smell	100	22 (7.5)	−0.41	69 (69)	17 (17)	15 (15)
Tactile	100	67 (19.8)	−0.89	55 (55)	23 (23)	23 (23)
Proprioception	99	23 (9.2)	−1.01	54 (54)	18 (18)	28 (27)
Postural Control	99	28 (6.7)	−0.83	63 (63)	25 (25)	12 (11)
Motor Control	99	25 (10.6)	−0.95	59 (59)	17 (17)	24 (23)
Social–Emotional	99	42 (11.2)	−0.98	52 (52)	23 (23)	25 (24)
Modulation	100	127 (34.2)	−0.77	58 (58)	27 (27)	16 (16)
Discrimination	98	132 (37.4)	−0.90	53 (53)	28 (28)	18 (17)

**Table 3:** MANCOVA results for mean scores on the five sensory, three motor, and social–emotional sections with gender, age, reported anxiety, currently receiving psychological services, and the total number of reported diagnoses.

Variable	Wilks’Λ	*F*	*η* ^2^
Age	0.907	*F*(9, 72) = 0.8, *p* = 0.602	0.093
Gender	0.779	*F*(9, 72) = 2.3, *p* = 0.027	0.221
Anxiety	0.930	*F*(9, 72) = 0.6, *p* = 0.793	0.070
Current psychological services	0.894	*F*(9, 72) = 0.9, *p* = 0.493	0.106
Number of reported diagnoses	0.812	*F*(9, 72) = 1.9, *p* = 0.074	0.188

**Table 4:** Paired *t*-tests with child and adult mean items scores for the total scores and sensory, motor, and social–emotional subscores.

Variable	Child Mean Item Score *m* (*sd*)	Adult Mean Item Score *m* (*sd*)	Paired Difference *m* (*sem*)	*t*	df	*p* (2-Tailed)
Total	1.7 (0.25)	1.5 (0.31)	0.22 (0.037)	5.804	95	<0.001
Visual–Spatial	1.6 (0.33)	1.4 (0.32)	0.15 (0.042)	3.712	98	<0.001
Auditory	1.8 (0.48)	1.7 (0.56)	0.09 (0.064)	1.467	97	0.146
Movement	1.7 (0.24)	1.4 (0.32)	0.26 (0.039)	6.707	96	<0.001
Taste and Smell	1.6 (0.49)	1.4 (0.39)	0.17 (0.056)	3.096	97	0.003
Tactile	1.7 (0.36)	1.5 (0.34)	0.20 (0.044)	4.620	97	<0.001
Motor	1.8 (0.39)	1.6 (0.41)	0.18 (0.052)	3.461	96	0.001
Social–Emotional	1.9 (0.31)	1.7 (0.42)	0.20 (0.051)	4.012	96	<0.001

## Data Availability

Data are available on request from the authors.
